# LOXL2 Expression Status Is Correlated With Molecular Characterizations of Cervical Carcinoma and Associated With Poor Cancer Survival via Epithelial-Mesenchymal Transition (EMT) Phenotype

**DOI:** 10.3389/fonc.2020.00284

**Published:** 2020-03-06

**Authors:** Canhui Cao, Shitong Lin, Wenhua Zhi, Cordelle Lazare, Yifan Meng, Ping Wu, Peipei Gao, Juncheng Wei, Peng Wu

**Affiliations:** ^1^Cancer Biology Research Center (Key Laboratory of the Ministry of Education), Tongji Hospital, Tongji Medical College, Huazhong University of Science and Technology, Wuhan, China; ^2^Department of Gynecologic Oncology, Tongji Hospital, Tongji Medical College, Huazhong University of Science and Technology, Wuhan, China

**Keywords:** LOXL2, molecular analyses, cervical cancer, EMT, APOBEC3 family genes, cancer survival

## Abstract

As molecular analyses based on high-throughput sequencing have developed, the molecular classification of cancer has facilitated clinical work. The aim of the present study was to identify a new potential therapeutic target for cervical carcinoma by molecular analyses. We firstly tested the LOXL2 expression pattern in 50 paired normal cervix and cervical carcinoma via qPCR and immunohistochemistry, and the LOXL2 expression pattern was found to be in accordance with public datasets from Gene Expression Omnibus (GEO). Then, we comprehensively rewired the 176 cervical carcinoma samples from The Cancer Genome Atlas (TCGA), subsequently clustered the samples into two groups corresponding to LOXL2 expression to determined the associations between LOXL2 expression status and molecular characterizations of cervical carcinoma. *In vitro* assays for further verifying the correlations in SiHa-shLOXL2 and HeLa-shLOXL2 cell lines. In this study, we found that LOXL2 highly expressed in carcinoma tissue, with 14 CpG islands of LOXL2 promoter that were significantly and negatively associated with its expression in cervical carcinoma. And there were notable correlations among LOXL2 expression status and molecular characterizations of cervical carcinoma, including diagnostic age, HPV A7 types, mRNA molecular clusters, miRNA molecular clusters, and DNA methylation molecular clusters et al. In addition, high LOXL2 expression was negatively correlated with lower tumor mutation density, especially in EP300, ERBB2, EGFR and NOTCH2, and was negatively correlated with lower expression of APOBEC3 family genes, such as APOBEC3A, APOBEC3B, APOBEC3D, and APOBEC3G. Furthermore, high LOXL2 expression was associated with poor overall (OS) and poor disease-free survival (DFS) in cervical carcinoma, and was associated with higher epithelial-mesenchymal transition (EMT) score, enrichment of extracellular matrix (ECM) signaling, the phenotype that was found to be associated with poor prognosis in cervical carcinoma from TCGA. Conversely, the ability of cell proliferation and cell migration were reversed in LOXL2 knock-down cervical cell lines via regulating the genes' expression of EMT phenotype *in vitro*. Overall, we demonstrated the correlation between LOXL2 expression status and cancer molecular characterizations of cervical carcinoma, and identified LOXL2 may serve as a therapeutic target for such carcinoma.

## Introduction

Cervical cancer accounts for 265,700 cancer-related deaths in women worldwide, more than any other gynecological tumor, with 527,600 new cases reported every year (1). The association between intratumor heterogeneity and cancer molecular function varies substantially in cervical cancer among different HPV types (2–4), which in turn provides insight into carcinogenesis beyond histological subtype. With the development of high-throughput sequencing using a large cervical carcinoma cohort, The Cancer Genome Atlas (TCGA) research network has identified three clusters according to mRNA expression, three clusters corresponding to reverse phase protein array (RPPA), six clusters based on miRNA expression, and two clusters based on copy number variation (CNV) data. This has highlighted molecular heterogeneity of cervical cancer not only from a histological perspective but also in relation to the molecular characterizations of cervical cancer. Based on gynecological and breast cancer data, previous pan-cancer molecular study showed that, among gynecological cancers, cervical cancer exhibits a high median mutation (5.3 mutations/mbp), high degree of hypermethylation, and expresses high immune marker signatures (5).

Lysyl oxidase-like 2 (LOXL2) is a member of the lysyl oxidase (LOX) family, which plays a critical role in catalyzing the formation of cross-links of elastin and collagen in the extracellular matrix (ECM) (6). LOXL2 has been implicated in promoting cancer cell proliferation (7), invasion (8), metastasis (9), and angiogenesis (10) in many cancer types. In addition, increased expression of LOXL2 is significantly associated with decreased survival in laryngeal squamous cell carcinoma (11), gastric cancer (12), pancreatic carcinoma (13), hepatocellular carcinoma (9), colon tumor (14), and basal-like breast carcinoma (15). Moreover, LOXL2 may serve as a target in the development of antibodies or inhibitors for cancer therapeutics (16, 17), as characterized in ClinicalTrials.gov NCT01323933.

In the current study, by rewiring TCGA cervical carcinoma data, including clinical information, HPV status, established molecular clusters, tumor mutation density, and APOBEC3 family gene expression, we demonstrated the correlation between LOXL2 expression status and the molecular characterizations of cervical carcinoma, and found that LOXL2 expression was negatively correlated with the expression of APOBEC3 family genes, especially APOBEC3A, APOBEC3B, APOBEC3D, and APOBEC3G *in vitro*. More importantly, increased expression of LOXL2 was significantly associated with decreased survival in cervical carcinoma, which was further associated with EMT phenotype *in vitro*.

## Materials and Methods

### Datasets

Integrated analyses were performed on a subset of 176 samples (the core-set) (Supplementary Table 1) from TCGA dataset (18), which included LOXL2 mRNA expression, clinical information of samples (age at initial pathologic diagnosis, lymph node status, pathology, and clinical stage), HPV status of samples (HPV categories, HPV integrated status, E6 ratio category), somatic genomic alterations density (non-silent mutation rate per MB, silent mutation rate per MB and total mutation rate per MB) of patients, molecular platform of established clusters (copy number clusters, methylation based clusters, mRNA based clusters, miRNA based clusters, uterine corpus endometrial-like (UCEC-like) based analysis and reverse phase protein array (RPPA) based clusters) and APOBEC family genes expression (APOBEC1, APOBEC3A, APOBEC3B, APOBEC3C, APOBEC3D, APOBEC3F, APOBEC3G, and APOBEC3H). All detail information was showed in Supplementary Table 1.

### Cells Culture

The human cervical cancer cell lines (SiHa and HeLa) were purchased from American Type Culture Collection (ATCC) and authenticated at China Center for Type Culture Collection (Wu Han University). All of the cells were cultured in DMEM (11965084, Gibco) containing 10% fetal bovine serum (FBS, SH30406.05, Hyclone) and antibiotics (100 U/mL penicillin and 100 μg/mL streptomycin, 15140163, Gibco) at 37°C with 5% CO_2_.

### RNA Interference

Cells were cultured in six-well plates for transfecting with MCS-shLOXL2-SV40-firefly-Luciferase-IRES-Puromycin lentiviral particles (GeneChem, Shanghai, China) and screened by puromycin (0.1 μg/mL, A1113803, Gibco) for 2 weeks. The sequences targeted the LOXL2 (Human NM_002318) gene by shRNA were 5′-GAAACCCTCCAGTCTATTATA-3′.

### Immunohistochemistry (IHC)

The cervical cancer samples and the paired adjacent normal cervix samples were obtained from The Biobank of Patients With Gynecologic Neoplasms (NCT01267851, ClinicalTrials.gov, https://clinicaltrials.gov/ct2/show/study/NCT01267851) in the department of gynecologic oncology of Tongji Hospital. The 4% paraformaldehyde fixed and paraffin-embedded sections (4 μm) were subjected to IHC assay according to the manufacturer's protocols. Briefly, The slides were put into the dewaxing solution I/II, 100% ethanol, 90% ethanol, 85% ethanol and 75% ethanol for 5 min respectively. Antigen retrieval was performed in Tris-EDTA (PH 9.0) using heat-induced protocol. And the slides were incubated at 4°C with LOXL2 antibody (GTX105085, GeneTex, 1:400) overnight. Staining detection was performed using DAB (Servicebo). Characteristics of patients were displayed in Supplementary Table 2.

### Immunofluorescence (IF)

Cells were seeded in glass coverslips that were placed in 24-well plates after 24 h for assay according to the protocols (http://media.cellsignal.com/www/pdfs/resources/product-literature/application-if-brochure.pdf). Briefly, cells were fixed by 4% paraformaldehyde for 30 min in room temperature, and penetrated by 0.3% Triton X-100 (T8200, Solarbio) for 30 min in 37°C before blocking step (5% bovine serum albumin (BSA) for 30 min in 37°C). Antibodies used for incubation were LOXL2 (GTX105085, GeneTex, 1:200) and Alexa Fluor Plus 488 (A32731, Invitrogen, 1:1000). Images were taken using Olympus microscope (BX53) equipped with FITC and DAPI filters.

### Western Blot

Cells were lysed in RIPA lysis buffer (P0013B, Beyotime) with protease inhibitor cocktail (04693132001, Roche). The protein was performed in SDS-PAGE (8012011, BioSci) and was electrically transferred onto PVDF membrane (10600023, GE). The PVDF membrane was incubated with diluted GAPDH (A10471, ABclonal, 1:2000), LOXL2 antibody (A4708, ABclonal, 1:1000), SNAI1 antibody (A12301, ABclonal,1:1000), E-Cadherin (GTX100443, 1:1000), N-Cadherin (A10206, ABclonal, 1:1000), Vimentin (3932, CST,1:1000), APOBEC3A (A12399, ABclonal, 1:400), APOBEC3B (A9010, ABclonal, 1:1000), APOBEC3D (A11648, ABclonal, 1:1000), APOBEC3G (A17199, ABclonal, 1:1000), Ki 67 (273091-1-AP, proteintech, 1:4000), Caspase 3 (A19654, ABclonal, 1:1000) at 4°C overnight. After incubation with HRP Goat anti-rabbit IgG (H+L) (AS014, ABclonal, 1:1000) at 37°C for 1 h, the PVDF membrane was performed using western bright ECL HRP substrate (K-12045-C20, Advansta) and analyzed by Image Lab (v4.1).

### RNA Extraction, PCR, and qRT-PCR

The total RNA of cells were extracted with Trizol reagent (15596026, Invitrogen) under the protocols of manufacturer. The cDNA was synthesized by HiScript II Q RT SuperMix (R223-01, Vazyme) according to the manufacturer's constructions. Bio-Rad CFX96 Real-Time System manager (C1000 Thermal Cycler) was used to detect the expression level of target genes using iTaq Universal SYBR Green (1725125, Bio-Rad), and GAPDH was used as the internal control. The specific primer sequences of targeted genes are displayed in Supplementary Table 3.

### Cell Viability Assay

Cells (*n* = 6,000) were seeded in the 96-well plates. The relative viability of cells were assayed by Cell Counting Kit-8 (CK04, Dojindo Laboratories) at each time point according to the protocols, which were measured by Multiskan Spectrum (Spectra Max190, Molecular Devices) with the wavelength of 450 nm. Assay was performed using six replications.

### Colony Formation Assays

Cells (*n* = 1,000) were seeded in six-well plates for 2 weeks to allow colony formation. Then, the plates were fixed with 4% paraformaldehyde for 10 min and stained with 0.1% crystal violet for 30 min.

### Transwell Chamber Assay

The transwell (3422, Corning Incorporated Costar, 8.0 μm pore size) was used to assess the ability of migration for cells according to the protocols (https://www.corning.com/catalog/cls/documents/protocols/Transwell_InstructionManual.pdf.). Cells (5 × 10^4^) were seeded in the upper chamber for incubation at each time point. Then the upper chamber was removed, fixed with paraformaldehyde for 10 min and stained with 0.1% crystal violet for 30 min. Finally, the upper chamber biofilm was cut off for further analysis.

### Wound Healing Assay

The same number of cells (4 × 10^5^) between the two groups were seeded in 6-well plates. Three horizontal and vertical scratches were made using 200 μL pipette tips in the plates, and the plates were washed with phosphate buffered saline (PBS) for three times. Then the cells were cultured in serum-free medium. The degree of cell migration to the blank was recorded by microscopy at 0 and 48 h, and analyzed with software Image J (v1.8.0).

### Bioinformatic Analyses

The correlation of DNA methylation and LOXL2 expression was performed by MEXPRESS (v2019) (19, 20) with default setting. All results were showed in Supplementary Table 4. Correlation analyses were performed in the Gene Expression Profiling Interactive Analysis (GEPIA) dataset (21). Correlation coefficient was operated by Pearson's test. All parameters were computed at the default setting. Survival plots of LOXL2 gene in cervical cancer was performed in GEPIA with default setting. LOXL2 mRNA expression in the groups of normal cervix and cervical cancer were conducted in the GEO expression profiling dataset (GSE63514, GSE7803, and GSE9750). LOXL2 correlated genes were performed by LinkedOmics (22) with default setting. Functional enrichment of the LOXL2 positively correlated genes was performed by Metascape (23) with FDR < 0.05, *r* > 0.3.

### EMT Score Analysis

The EMT score was downloaded from Supplementary Data of TCGA research network, and the EMT score algorism was computed as previously published (24). Briefly, the score was the value that the average expression of stromal genes minus the average expression of epithelial genes. Removed all unavailable data (NA) values from the calculation. Accordingly, a *t*-test and ANOVA test were applied to each comparison.

### Statistical Analyses

Data were presented as mean ± standard deviation (SD) or standard error of mean (SEM). Results were calculated by GraphPad Prism 6 (version 6.02) software. The Student's *t*-test, Mann-Whitney test, one-way ANOVA, Two-way ANOVA, log-rank test, Cox model or Chi-Square test as indicating in figure legends. The expression of APOBEC3 genes was shown with log2 of (RSEM (RNA-Seq by Expectation-Maximization)+1) according the TCGA research network. Mean and SD were shown. Single comparisons between the low- and high- LOXL2 groups were determined by Student's *t*-test. Kaplan–Meier curves were based on the log-rank test. The HR was performed using the Cox model. *P*-value < 0.05 was considered to be significant.

## Results

### LOXL2 Expression Profile in Cervical Cancer Tissues

**Figure 1:**
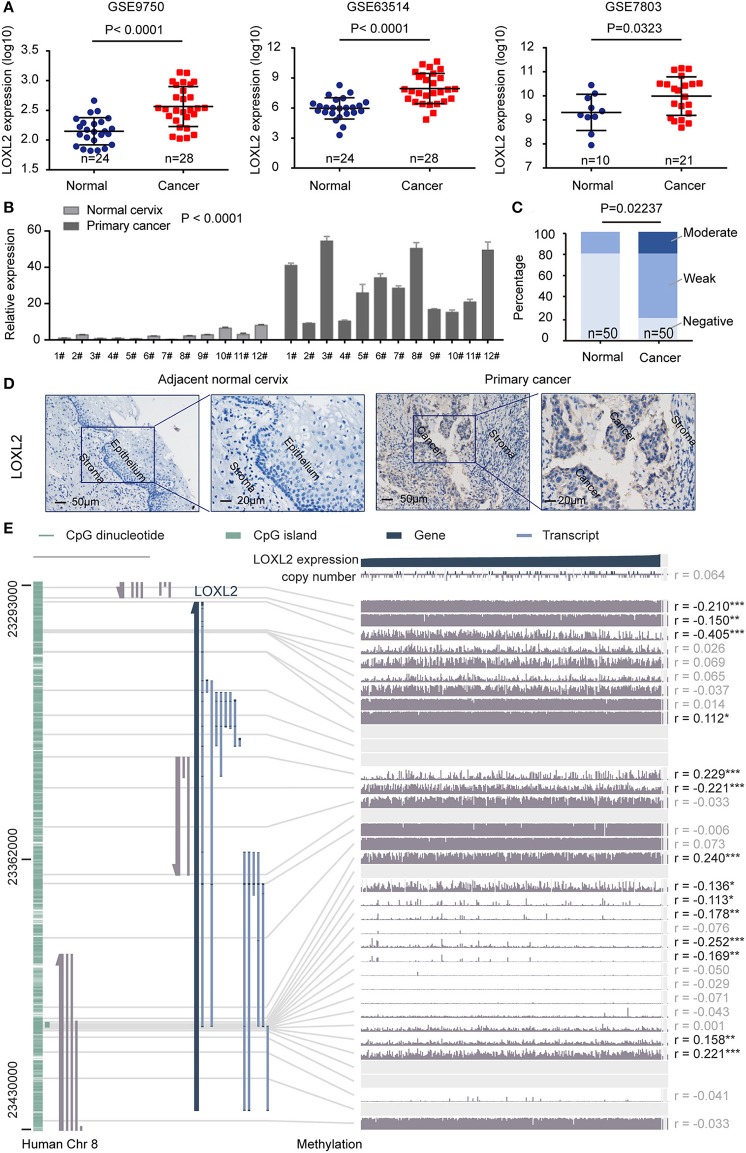
LOXL2 expression profile in cervical cancer tissues. **(A)** Normalized expression of LOXL2 in the normal cervix and primary cancer using three expression profiling data (GSE63514, GSE7803, and GSE9750). *P-*values were calculated by Student's *t*-test Mean and SD were shown. **(B)** qPCR of LOXL2 relative expression in paired adjacent normal cervix and cancer tissues. Mean and SEM (*n* = 3) were shown. *P-*values were calculated by Two-way ANOVA test. ^#^Means patients number. **(C)** Scoring of LOXL2 in the stained tissues. *P-*values were calculated by Chi-square test. **(D)** Representative images of IHC staining of LOXL2 in the adjacent normal cervix and primary cancer. **(E)** The correlation of LOXL2 expression and DNA methylation of LOXL2 promoter from MEXPRESS (19, 20), including 317 cervical squamous cell carcinoma and endocervical adenocarcinoma from TCGA datasets. The statistics (correlation coefficient (r) and *P-*value) on the right show how LOXL2 expression and DNA methylation of promoter were negatively correlated (* <0.05, ** <0.01, *** <0.001).

We first investigated the expression pattern of LOXL2 in the GEO dataset (Figure 1A) and in clinical samples of paired adjacent normal cervix and cancer tissues using quantitative polymerase chain reaction (qPCR) (Figure 1B) and immunohistochemical (IHC) assays (Figures 1C,D and Supplementary Figure 1). Integrative analyses of LOXL2 expression were conducted in three GEO expression profile datasets (i.e., GSE63514, GSE7803, and GSE9750), which included transcriptomic profiles of normal cervix and primary cancer tissue. We found that the average expression of LOXL2 was elevated in primary cancer compared with normal cervix tissue. Additionally, IHC staining demonstrated that 80% of adjacent normal cervix samples vs. 20% of primary cancer samples showed negative staining, 20% of normal samples vs. 60% of cancer samples showed weak staining, and 20% of cancer samples showed moderate staining (Figures 1C,D, and Supplementary Figure 1). In addition, LOXL2 also expression in the adjacent stroma of cervical cancer slide (Figure 1D, and Supplementary Figure 1). These results demonstrated that LOXL2 expression was significantly increased in cervical carcinoma.

To demonstrate the correlation between LOXL2 expression and DNA methylation of the LOXL2 promoter, we performed integrated visualization of LOXL2 expression and DNA methylation data in MEXPRESS (19, 20) based on 317 cervical squamous cell carcinomas and endocervical adenocarcinomas from TCGA (Figure 1E). We identified 14 CpG islands of LOXL2 (i.e., cg20981791, cg20142986, cg24531955, cg17804498, cg09535960, cg04028450, cg10090386, cg09042448, cg18233786, cg22996912, cg00558156, cg25074071, cg05365729, and cg04259752) that were significantly and negatively associated with LOXL2 gene expression in cervical carcinoma (Supplementary Table 4).

### Multiplatform Integrative Analyses of Cervical Carcinoma Data Clustered by LOXL2 Expression Status

**Figure 2:**
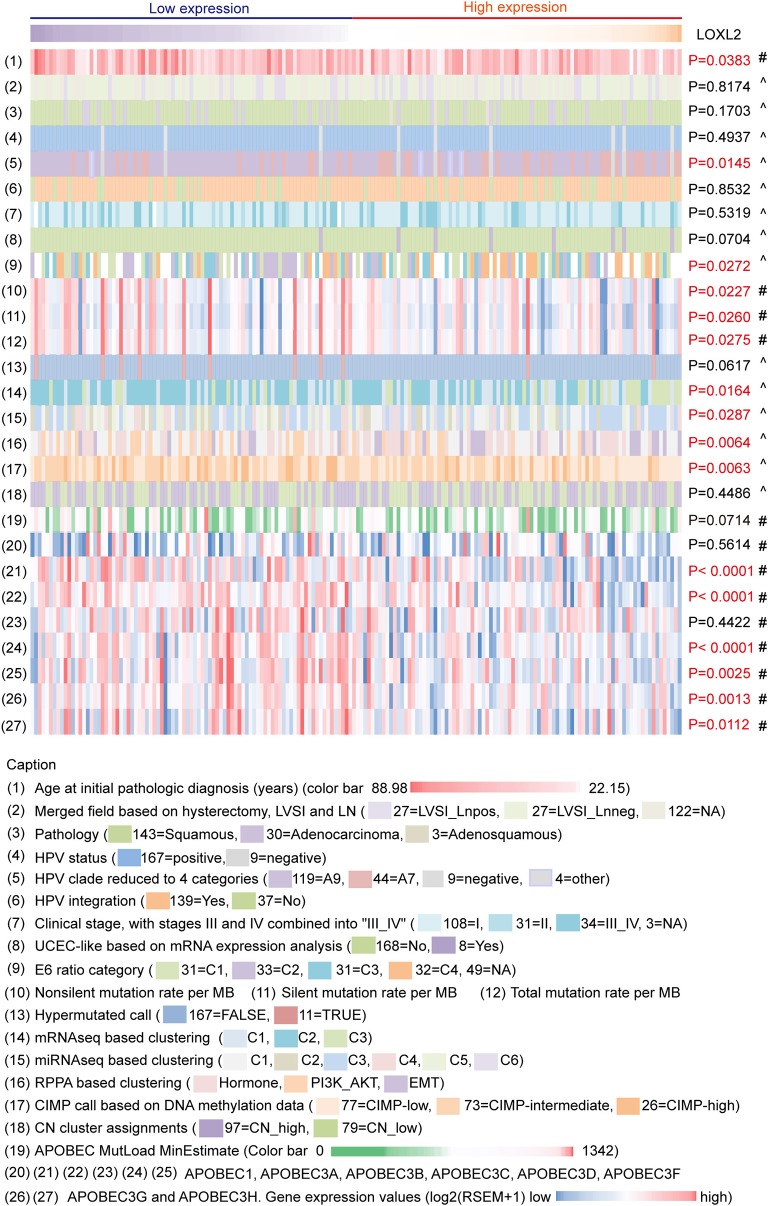
Multiplatform integrative analyses of cervical cancer data clustered by LOXL2 expression. 176 core-set cervical cancer samples were clustered into two groups according to LOXL2 mRNA expression (low LOXL2 expression, high LOXL2 expression). Clinical information, HPV genotypes of samples, somatic genomic alterations, molecular platform features corresponding to the TCGA clustering and the APOBEC3 family genes expression were showed by color. Color captions were shown in the bottom. Each column represented a sample. ^#^*P-*values were calculated by Mann–Whitney test. ^∧^*P* values were calculated by Chi-Square Test. Red *P-*values indicate *P* < 0.05.

We integrated the LOXL2 mRNA expression levels, clinical information of patients (age at initial pathologic diagnosis, lymph node status, pathology, and clinical stage), HPV status of samples (HPV categories, HPV integrated status, E6 ratio category), somatic genomic alteration densities (non-silent, silent, and total mutation rates per MB) of patients, molecular platform of established clusters (copy number clusters, methylation-based clusters, mRNA-based clusters, miRNA-based clusters, UCEC-like-based analysis, and RPPA-based clusters) corresponding to TCGA clustering to analyze the molecular characterizations based on LOXL2 status in cervical carcinoma (Figure 2, Supplementary Table 1, Supplementary Figure 2 and Methods). Samples were ordered by LOXL2 expression and classified into two clusters, i.e., low-LOXL2 cluster and high-LOXL2 cluster.

Many studies have identified the important role of APOBEC3 genes in HPV-related cancers (18, 25). There was direct evidence that APOBEC3 edited HPV DNA (25) and there were notable APOBEC mutagenesis patterns in cervical carcinoma sequencing (18). Therefore, we also integrated the APOBEC family (APOBEC1 APOBEC3A, APOBEC3B, APOBEC3C, APOBEC3D, APOBEC3F, APOBEC3G, and APOBEC3H) mRNA expression of 176 core-set cervical carcinoma samples for multiplatform integrative analyses (Figure 2, Supplementary Table 1, Supplementary Figure 2).

For clinical information, we found that the mean age at initial pathological diagnosis differed between the two clusters (*P* = 0.0383). For HPV infection, HPV A7 types were enriched in the high-LOXL2 cluster (*P* = 0.0145), consistent with the enrichment of HPV A7 type in low-keratin and adenocarcinoma clusters reported in TCGA (18). In addition, the E6 ratio category was significantly different (*P* = 0.0272), especially C2 in the low-LOXL2 cluster and C4 in the high-LOXL2 cluster (E6 category (31 = C1, 33 = C2, 31 = C3, 32 = C4, 49 = NA), Supplementary Table 1). However, HPV integration showed no correlation with LOXL2 expression status (*P* = 0.8532). For somatic mutation density divided by genome length (MB), the mean densities of the non-silent (*P* = 0.0227), silent (*P* = 0.0260), and total mutation rates (*P* = 0.0275) were correlated with LOXL2 expression. For molecular platform features, the mRNA-Seq (*P* = 0.0164), miRNA-Seq (*P* = 0.0287), and DNA-methylation clusters (*P* = 0.0063) based on unsupervised hierarchical clustering were significantly correlated with LOXL2 expression status. For the APOBEC3 family genes, the mean expression levels of APOBEC3A (*P* < 0.0001), APOBEC3B (*P* < 0.0001), APOBEC3D (*P* < 0.0001), APOBEC3F (*P* = 0.0025), APOBEC3G (*P* = 0.0013), and APOBEC3H (*P* = 0.0112) were different between the LOXL2 clusters.

### Mean Diagnostic Age and Tumor Mutation Density Were Negatively Correlated With LOXL2 Expression Cluster

**Figure 3:**
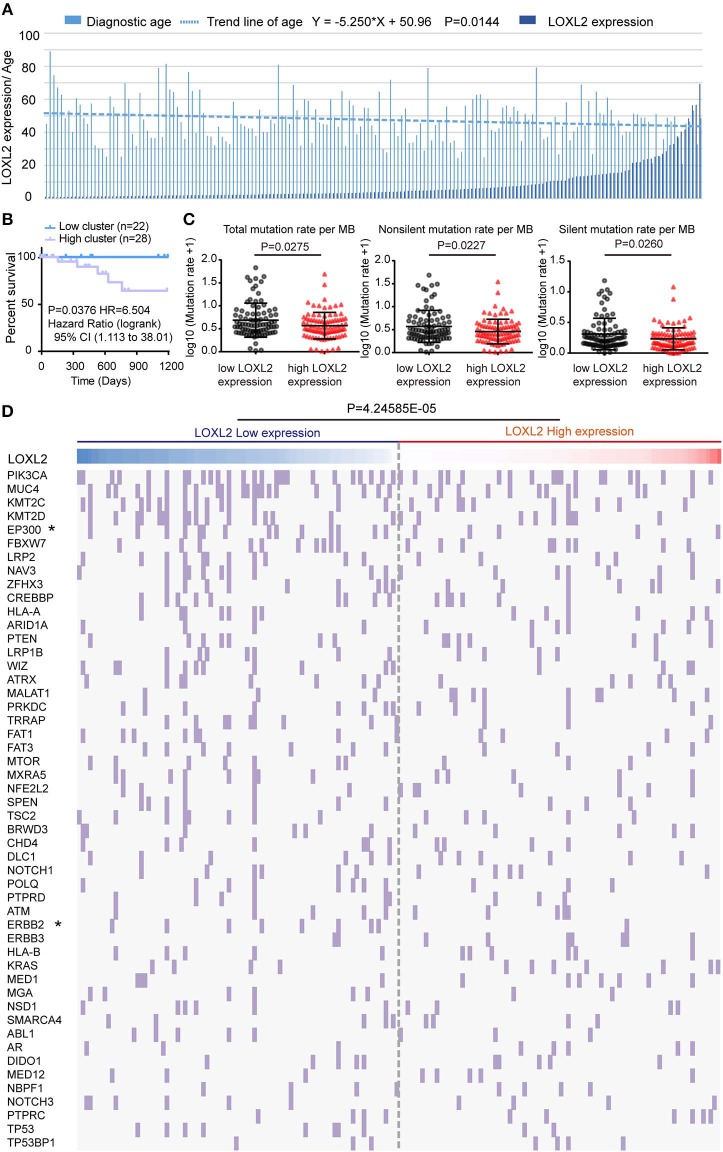
The mean diagnostic age and tumor mutational density were negatively correlated with LOXL2 expression cluster. **(A)** Clustered column of LOXL2 expression and the diagnostic age of 176 core-set cervical cancer samples, which was ordered by the LOXL2 expression. *P-*values were calculated by linear regression about log10 of LOX2 expression and the diagnostic age. Equation was shown above. **(B)** Three years Kaplan–Meier survival curve between the LOXL2-high and -low groups corresponding to diagnostic age 20 to 39. **(C)** Scatter plots of samples' somatic genomic alterations intensity in the two LOXL2 subgroups, which represent total, non-silent, silent mutation rate per MB. Y axis shows the log10 of the mutation rate per MB. Mean and SD were shown. *P-*values were calculated by Mann-Whitney test. **(D)** 176 core-set cervical cancer samples (columns) arranged by LOXL2 expression, tumor mutation density corresponding to top 50 somatic genomic alteration genes (rows) of patients in cervical cancer were calculated. Each light purple column represents a somatic genomic alteration. *P-*values were calculated by Chi-square test.

To identify the clinical characteristics associated with LOXL2 expression in cervical cancer, we arranged LOXL2 expression and diagnostic age of the 176 core-set samples, which were ordered by LOXL2 expression (Figure 3A). We then compared the mean age at initial pathological diagnosis between the two clusters (Supplementary Figure 3A), i.e., 49.64 ± 1.462 in low-LOXL2 cluster vs. 45.55 ± 1.305 in high-LOXL2 cluster and found that diagnostic age was negatively associated with LOXL2 expression status. According to the latest worldwide statistics, cervical cancer is the second leading cause of cancer deaths in the 20- to 39-year age group, with nine cases reported per week (26). Thus, we compared the three-year OS for the 20- to 39-year age group between the two clusters, with poorer outcomes found for the high-LOXL2 cluster (Figure 3B, Supplementary Table 5).

Cervical carcinoma exhibits a high tumor mutation burden (TMB) and microsatellite instability (MSI) stable carcinoma (27). Thus, we compared the mean somatic genomic mutation density between the two clusters (Supplementary Table 6). We found that the mean densities of the total, non-silent, and silent mutation rates (Figure 3**C**) were higher in the low-LOXL2 cluster than in the high-LOXL2 cluster.

By ordering the 176 core-set cervical cancer samples by LOXL2 expression, we found that the somatic alteration density was high in the low-LOXL2 cluster when considering the top 50 (*P* = 4.24585E-05) (Figure 3D), top 1-10 (*P* = 3.40597E-05), top 21-30 (*P* = 0.048011) (Supplementary Figure 3B, and Supplementary Table 6) mutation genes in the dataset. Furthermore, the EP300 (*P* = 0.0289), ERBB2 (*P* = 0.0166), EGFR (*P* = 0.0166), and NOTCH2 (*P* = 0.0113) mutations were significantly associated with both clusters (Table 1 and Supplementary Table 6).

**Table 1:** Four mutation genes that were significant associated with the LOXL2 clusters.

**Characteristics**	**Total patients**		**LOXL2 low expression**		**LOXL2 high expression**		**P value**
	***N*** **=** **176 (No. %)**		***N*** **=** **88 (No. %)**		***N*** **=** **88 (No. %)**		
**Genes**	**Mutation**	**Non-mutation**	**Mutation**	**Non-mutation**	**Mutation**	**Non-mutation**	
EP300	19 (10.80%)	157 (89.20%)	14 (15.91%)	74 (84.09%)	5 (5.68%)	83 (94.32%)	0.0289
ERBB2	9 (5.11%)	167 (94.89%)	8 (9.09%)	80 (90.90%)	1 (1.14%)	87 (98.86%)	0.0166
EGFR	9 (5.11%)	167 (94.89%)	8 (9.09%)	80 (90.90%)	1 (1.14%)	87 (98.86%)	0.0166
NOTCH2	8 (4.55%)	168 (95.45%)	8 (9.09%)	80 (90.90%)	0 (0%)	88 (100%)	0.0113

*P-values were calculated by Chi-Square Test*.

### APOBEC3 Family Gene Expression Levels Were Negatively Correlated With LOXL2 Status in Cervical Carcinoma

**Figure 4:**
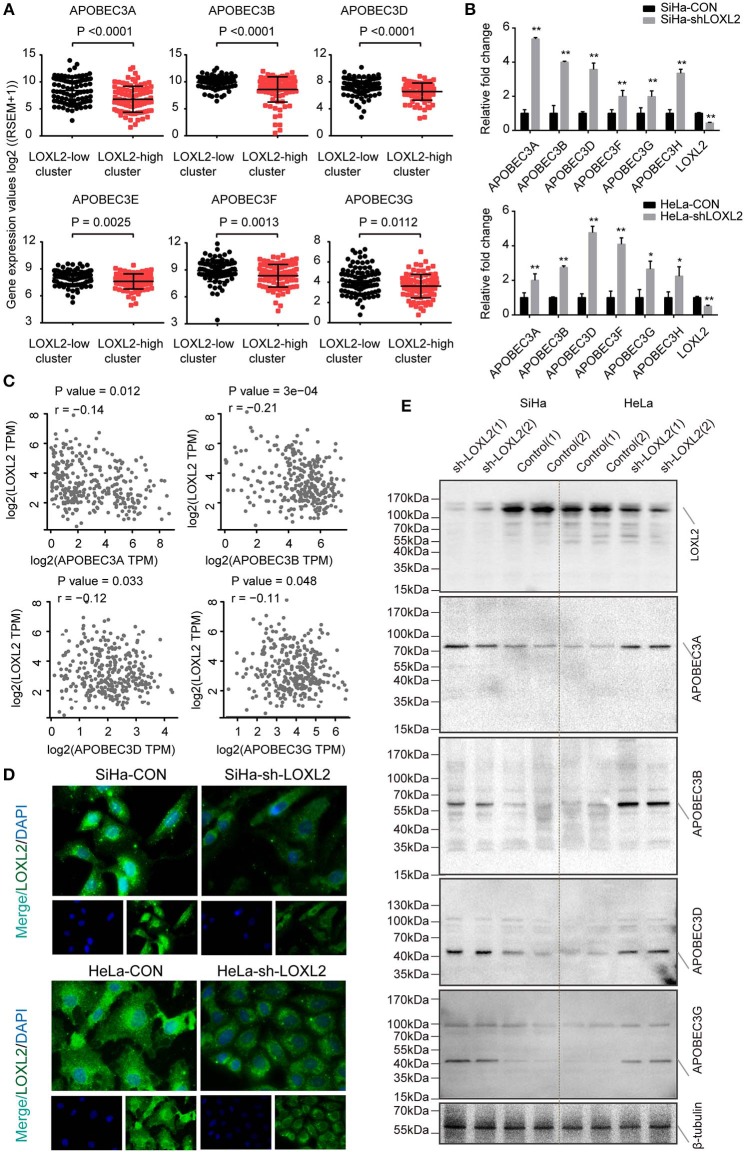
The expression of APOBEC3 family genes negatively correlated with the LOXL2 status in cervical cancer. **(A)** Scatter plots of APOBEC3 family mRNA expression in the two groups clustered by LOXL2 expression in 176 core-set cervical cancer samples. Y axis shows the log2 of (RSEM (RNA-Seq by Expectation-Maximization)+1). Mean and SD were shown. *P-*values were calculated by Student's *t*-test. **(B)** qPCR of APOBEC3 family genes in SiHa from CON and sh-LOXL2 (*n* = 3). Mean and SEM were shown. *P-*values were calculated by Student's *t*-test. (* <0.05, ** <0.01) **(C)** Pearson correlation analysis of APOBEC3 family genes as APOBEC3A, APOBEC3B, APOBEC3D, and APOBEC3G with that of LOXL2 expression in TCGA dataset from GEPIA (21). *P-*value and Pearson correlation coefficient (r) were shown. **(D)** Representative images of IF assay in SiHa and HeLa cell lines. **(E)** Western blot assay of LOXL2, APOBEC3A, APOBEC3B, APOBEC3D, and APOBEC3G in SiHa and HeLa after transfected with sh-Con or sh-LOXL2. β-tubulin served as the loading control.

Previous studies have indicated that APOBEC3 family genes play a key role in innate immunity (28, 29), HPV associated carcinogenesis (30), and development of chemoresistance in cancer (31, 32). By clustering the 176 core-set samples into two groups, we found that the mean expression of APOBEC3 family genes was higher in the low-LOXL2 cluster (Figure 4A), including APOBEC3A (8.384 ± 0.2478 in low cluster vs. 6.80 ± 0.2578 in high cluster), APOBEC3B (9.904 ± 0.1226 in low cluster vs. 8.588 ± 0.2501 in high cluster), APOBEC3D (7.273 ± 0.1367 in low cluster vs. 6.547 ± 0.1360 in high cluster), APOBEC3F (7.988 ± 0.09133 in low cluster vs. 7.597 ± 0.08861 in high cluster), APOBEC3G (8.929 ± 0.1288 in low cluster vs. 8.333 ± 0.1326 in high cluster), and APOBEC3H (4.163 ± 0.1426 in low cluster vs. 3.617 ± 0.1237 in high cluster). Our *in vitro* experiment also verified these associations at the mRNA level (Figure 4B).

To validate the expression patterns of APOBEC3 genes and LOXL2, we carried out correlation analysis using the GEPIA dataset, which included expression profiles of 306 cervical cancer tissues (21). Results showed that APOBEC3A (*r* = −0.14, *P* = 0.012), APOBEC3B (*r* = −0.21, *P* = 3e−4), APOBEC3D (*r* = −0.12, *P* = 0.033), and APOBEC3G (*r* = −0.11, *P* = 0.048) were statistically correlated with LOXL2 in cervical cancer (Figure 4C) and the APOBEC3C, APOBEC3F and APOBEC3H were not correlated (Supplementary Figure 4), the correlation coefficient seem to not be significantly correlated. Therefore, we performed the western blot assay in LOXL2 RNA-interference (Figure 4D) cells for further analysis. We found that the LOXL2-interference resulted in an increase in APOBEC3A, APOBEC3B, APOBEC3D, and APOBEC3G protein levels (Figure 4E) in the cervical cancer cells.

### High Levels of LOXL2 Were Associated With Poor Cancer Survival, Which Was Associated With EMT Phenotype

**Figure 5:**
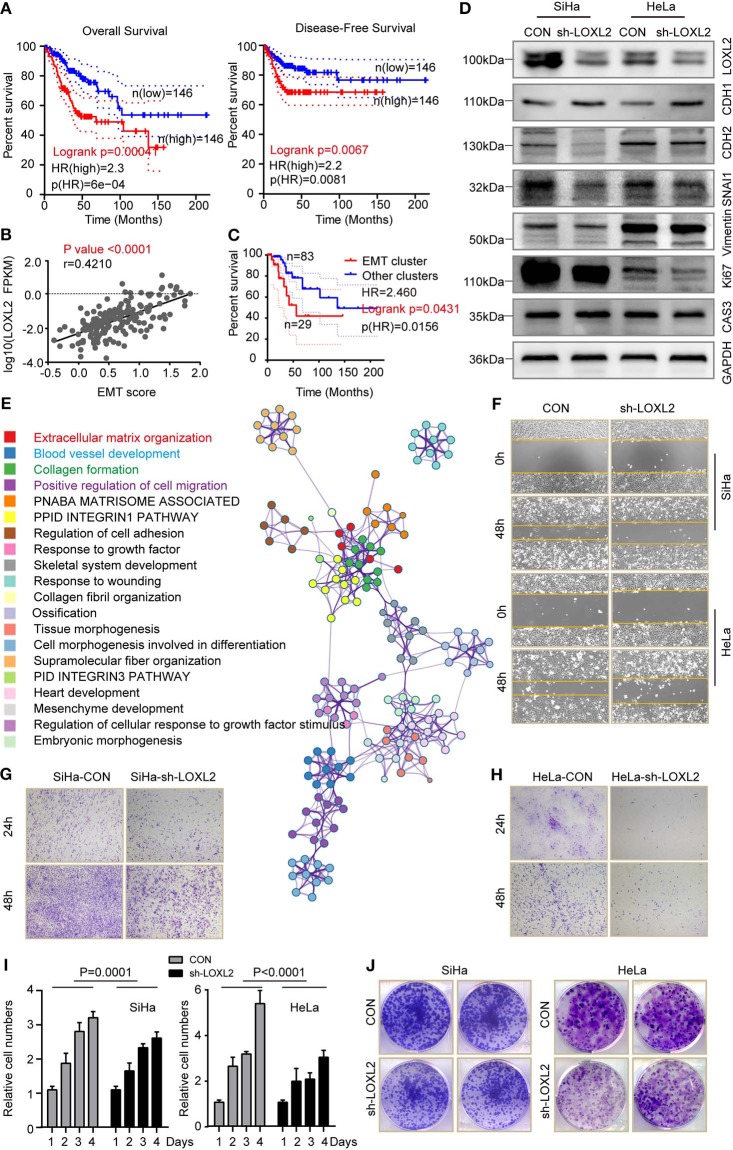
High level of LOXL2 was associated with poor cancer survival, which was associated with EMT phenotype. **(A)** Overall survival and disease-free survival were compared between the LOXL2-high and -low groups corresponding to LOXL2 expression. **(B)** Spearman correlation analysis of LOXL2 expression with that of normalized EMT score in 176 core-set cervical cancer samples. *P-*value and Pearson correlation coefficient (r) were shown. **(C)** Kaplan–Meier survival curve comparing overall survival across RPPA clusters (EMT cluster and other clusters) using 115 data from 176 core-set cervical cancer samples. Kaplan–Meier curves were based on the log-rank test. The HR was performed using the Cox model. **(D)** Western blot assay of LOXL2, E-Cadherin (CHD1), N-Cadherin (CHD2), SNAI1, Vimentin, Ki67 and Caspase 3 in SiHa and HeLa after transfected with sh-Con or sh-LOXL2. GAPDH served as the loading control. **(E)** Enrichment terms of LOXL2 positive correlated genes and network of enriched terms (Colored by cluster of enriched terms). Representative images of wound healing assay **(F)**, cellular migration assay **(G,H)**, cell viability assay **(I)** and colony formation assay **(J)** of SiHa and HeLa in CON or sh-LOXL2 groups at 0 and 48 h time points. Each assay was displayed more than three times.

As LOXL2 expression status was correlated with molecular characterizations of cervical cancer, we performed cancer survival analysis for the two clusters. High LOXL2 expression was found to be significantly correlated with poor OS (HR = 2.3, p(HR) = 6e−04) (Figure 5A and Supplementary Figure 5A) and with poor DFS (HR = 2.2, p(HR) = 0.0081) (Figure 5A) in the 292 cervical cancer TCGA dataset from GEPIA (21). Given that RPPA-based clustering was correlated with LOXL2 expression status, especially enrichment of the EMT cluster in the high-LOXL2 cluster, we performed correlation analysis between LOXL2 expression and EMT score and found that they were positively correlated (*P* < 0.0001, *r* = 0.4210) (Figure 5B). In addition, the EMT group exhibited poorer outcome (HR = 2.5, p(HR) = 0.0156) in OS (Figure 5C), which was well-established in TCGA network (18). Thus, we hypothesized that high LOXL2 expression showing poor cancer survival may be associated with the EMT phenotype.

To further explore the links between LOXL2 and the EMT phenotype, we detected protein levels *in vitro*. Results showed a decrease in N-cadherin (CHD2), SNAI1, and vimentin, and an increase in E-cadherin (CHD2) in the SiHa-sh-LOXL2 and HeLa-sh-LOXL2 cells (Figure 5D). Furthermore, these results showed the same tendency of LOXL2 positively correlated genes in cervical carcinoma (Figure 5E, Supplementary Figures 5B–D, and Supplementary Table 7), which was associated with ECM structural constituents (Supplementary Tables 8, **9**). In addition, cell diminished in the SiHa and HeLa LOXL2-interfering cells (Figures 5F-H and Supplementary Figure 5E) and the cell viability (Figure 5I) and colony formation assays (Figure 5J) revealed that cell proliferation was attenuated after LOXL2 silencing. To further investigate the role of LOXL2 in cell proliferation, we performed Ki 67 and Caspase 3 blotting in LOXL2-interfering cells. Result showed that LOXL2 knockdown blocked cell division instead of promoting cell death in cervical cancer (Figure 5D, Supplementary Figure 6).

## Discussion

Precise classification of cervical carcinoma in combination with multiplatform profiling can help to identify novel clinical and molecular associations in functional phenotypes, as well as predict disease outcome and guide selection of tumor-specific therapeutic approaches (18, 33). By rewiring the TCGA and GEO datasets with multiplatform profiling corresponding to LOXL2 expression status, we identified the correlation between LOXL2 and the molecular clusters established in TCGA. And the different features in the same patient indicated that LOXL2 expression status may reflect a combination of tumor heterogeneity and tumor-associated functional phenotype and represent an opportunity to subtype stratified clustering.

The U.S. Food and Drug Administration (FDA) has officially approved Keytruda (pembrolizumab) for cervical cancer patients with recurrent or metastatic disease during or after chemotherapy. However, previous cervical cancer clinical trials have reported relatively low overall response rates (ORR) with this drug (34). It has been reported that PD-L1 is expressed in <50% of samples (35), except for the current predictive value of PD-L1 immunohistochemistry, the other biomarkers should be assessed. Here, LOXL2 clustering identified different mutational density between the two clusters, especially for the EP300, ERBB2, EGFR, and NOTCH2 genes. As high TMB is considered to be an emerging biomarker of sensitivity to PD-1 and PD-L1 blockades and immune checkpoint inhibitors (27), LOXL2 clustering may be a new biomarker for screening immunotherapy, accompanied by PD-1 or PD-L1 expression in clinical triage, which needs subsequent research.

In innate immunity, the role of APOBEC3 genes in HPV-related cancer is presumed to be an aberrant trigger and/or dysregulation that results in somatic mutation observed in cervical cancer (31). For the different mean expression patterns of APOBEC3 genes between the two LOXL2 clusters observed in our research and their negative correlation in the SiHa and HeLa cell lines, we tentatively put forward LOXL2 may be a trigger of APOBEC3 gene dysregulation in cervical cancer. Furthermore, HPV A7 type, a type including HPV 18, 39, 45, 59, 68 subtypes, was observed in the high-LOXL2 cluster. According to the lasted TCGA clustering (18), most HPV clade A7 samples were with low CpG island hypermethylated (CIMP-low). In our clustering, HPV A7 type (*P* = 0.0145) and the CIMP-low group (*P* = 0.0063) was enriched in the high-LOXL2 cluster. The high expression of LOXL2 may be associated with the infection of type of HPV A7 and CpG island methylation of genes, which is worthy of further investigation.

For predicting potency, the high-LOXL2 cluster showed younger mean diagnostic age, with poorer cancer outcome in the 20–39 age range. In addition, high LOXL2 expression was significantly correlated with poor OS and DFS in cervical cancer. However, the mortality of cervical cancer is related to age, older women were seen to have the highest mortality rates (36). In our study, we found that high-LOXL2 cluster showed younger mean diagnostic age, while high-LOXL2 cluster showed poor cancer survival, which meant the poor prognosis in high-LOXL2 cluster was not completely on account of age at initial pathological diagnosis. Therefore, we analyzed the poor prognosis of high-LOXL2 cluster in cervical cancer, such as EMT phenotype in cancer progression. It is well-known that LOXL2 is involved in SNAI1 interaction and CDH1 repression for EMT induction (37). We found that the expression of LOXL2 was positively correlated with EMT phenotype in cervical cancer, and the proliferation and migration of cells were attenuated after LOXL2 silencing. These results contributed to the prediction potency of LOXL2 in cervical cancer, in accordance with the finding that the EMT phenotype is significantly correlated with poor prognosis in cervical carcinoma (18).

In many cancer types, EMT became aberrantly activated during tumor invasion and metastasis or sped up the processes of metastatic colonization (38). It was reported that overexpression of LOXL2 drove EMT via upregulating the expression of SNAIL (SNAI1/2), ZEB (ZEB1/2) via IRE1-XBP1 signaling pathway or FAK/SRC signaling pathway in cancer cells (39, 40). Furthermore, there was an autocrine feedback loops among ESRP1, HAS2, CD44, and ZEB1 in the aberrant activation of EMT (41), which acted in the dynamics of EMT in cancer metastasis. In addition, LOXL2 was also involved in the process of extracellular matrix (ECM) remodeling (42). ECM contributes to EMT and enhances cellular invasion via altering mechanical stiffness of the ECM and weakening the cell to cell adhesion (43, 44). In turn, LOXL2 induced aberrant activation of EMT crosslinked collagen in ECM remodeling for tumor microenvironment (41). Furthermore, LOXL2, as a copper-dependent enzyme, could be inhibited by copper chelation agents, such as penicillamine, trientine, disulfiram, clioquinol, or tetrathiomolybdate (TM), which could be used in cancer treatment (45, 46). Although TM has been demonstrated as controversial observations in cancer clinical trials, especially in breast cancer, the phase II trial of breast cancer demonstrated that women who had a high risk of recurrence would get benefit from TM treatment via changing the tumor microenvironment to prevent metastasis (47). There was potential value of copper chelation agents in cervical cancer for further research.

It should be noted that, in this study, we has examined only the expression pattern of LOXL2 in cervical carcinoma or paired normal tissue, the specific application and the significance in clinical samples should be further accessed, such as routine immunostaining. And there was specificity of LOXL2, high LOXL2 expression both in epithelial and stroma, which could be conducive to the epithelial–stromal cross talk and the malignant progression of cervical cancer (48, 49). We focused on the correlation between characterizations of carcinoma and LOXL2 expression status in cervical carcinoma, however, not all the results are significantly meaningful, especially in the stage, grade or histologic subtype of carcinoma, which is the evaluating indicator of carcinoma in clinical work. The correlation may reflect some part of molecular characterizations in cervical carcinoma. In addition, higher mutation burden is considered to be characteristic of high aggressive and mesenchymal cancers (50), there was a contradiction between TMB and LOXL2 expression status in our study. For low mutation density in high LOXL2 group, with poor outcome in high LOXL2 group, we tended to use the LOXL2 clustering as a biomarker for screening candidates for immunotherapy.

In summary, our results supported the relevance of LOXL2 expression status in multiplatform integrative analyses, prognosis of tumor mutation density, and survival prediction in cancer. And we showed the clinical and molecular associations as well as functionally altered features of LOXL2 expression, which may drive carcinogenesis and potentially serve as a therapeutic target in cervical carcinoma.

## Data Availability Statement

The datasets analyzed in this study can be found in the The Cancer Genome Atlas (https://portal.gdc.cancer.gov/).

## Ethics Statement

The studies involving human participants were reviewed and approved by Medical Ethics Committee of Tongji Medical College, Huazhong University of Science and Technology. The patients/participants provided their written informed consent to participate in this study.

## Author Contributions

PeW designed the experiments and supervised the study. CC, SL, and WZ performed the experiments. CC, CL, and SL wrote the manuscript. PiW, YM, and PG contributed to samples' collection. JW and PeW for funding acquisition.

### Conflict of Interest

The authors declare that the research was conducted in the absence of any commercial or financial relationships that could be construed as a potential conflict of interest.
