# Supplementary material for: LOXL2 Expression Status Is Correlated With Molecular Characterizations of Cervical Carcinoma and Associated With Poor Cancer Survival via Epithelial-Mesenchymal Transition (EMT) Phenotype
Source: Front Oncol. 2020 Mar 6;10:284. doi: 10.3389/fonc.2020.00284 (PMC7067748; doi:10.3389/fonc.2020.00284)
Supplement: Supplementary file 1 [file Data_Sheet_1.DOCX]

**Supplementary Table S2. Characteristics of patients with cervical cancer.**


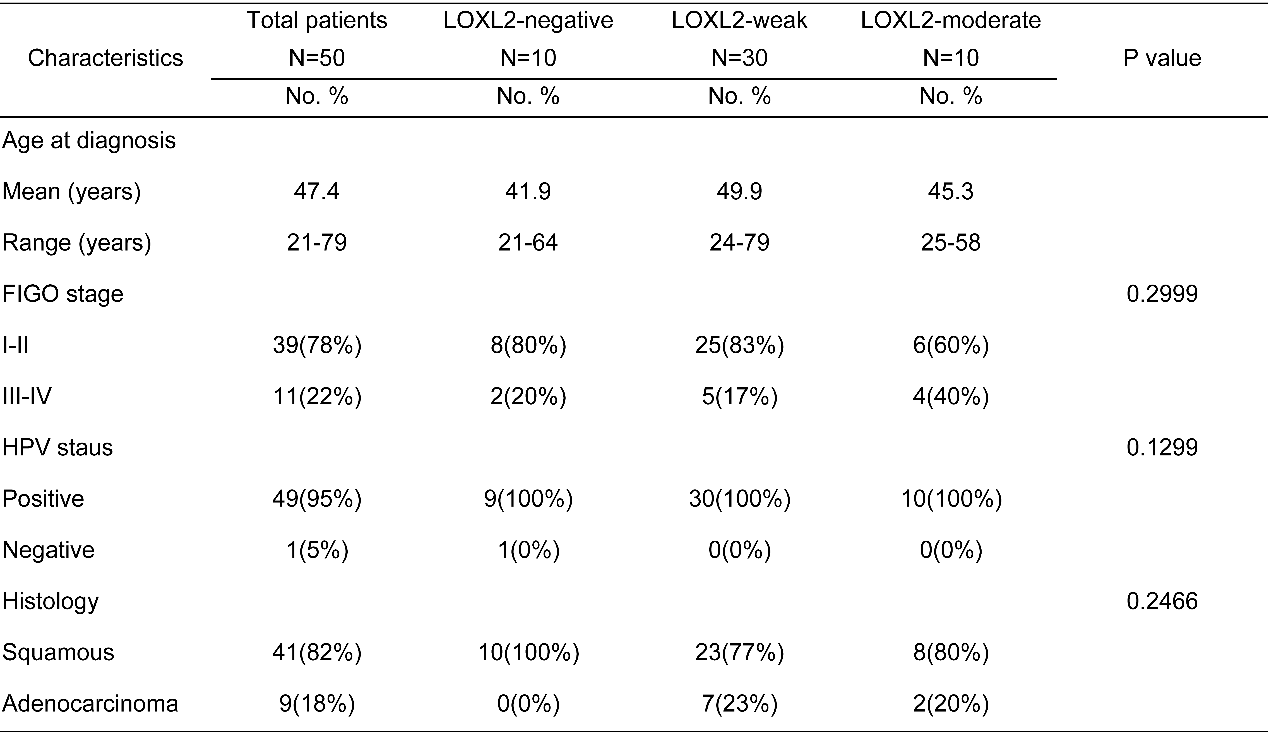


P values were calculated by Chi-Square Test.

**Supplementary Figure S1**


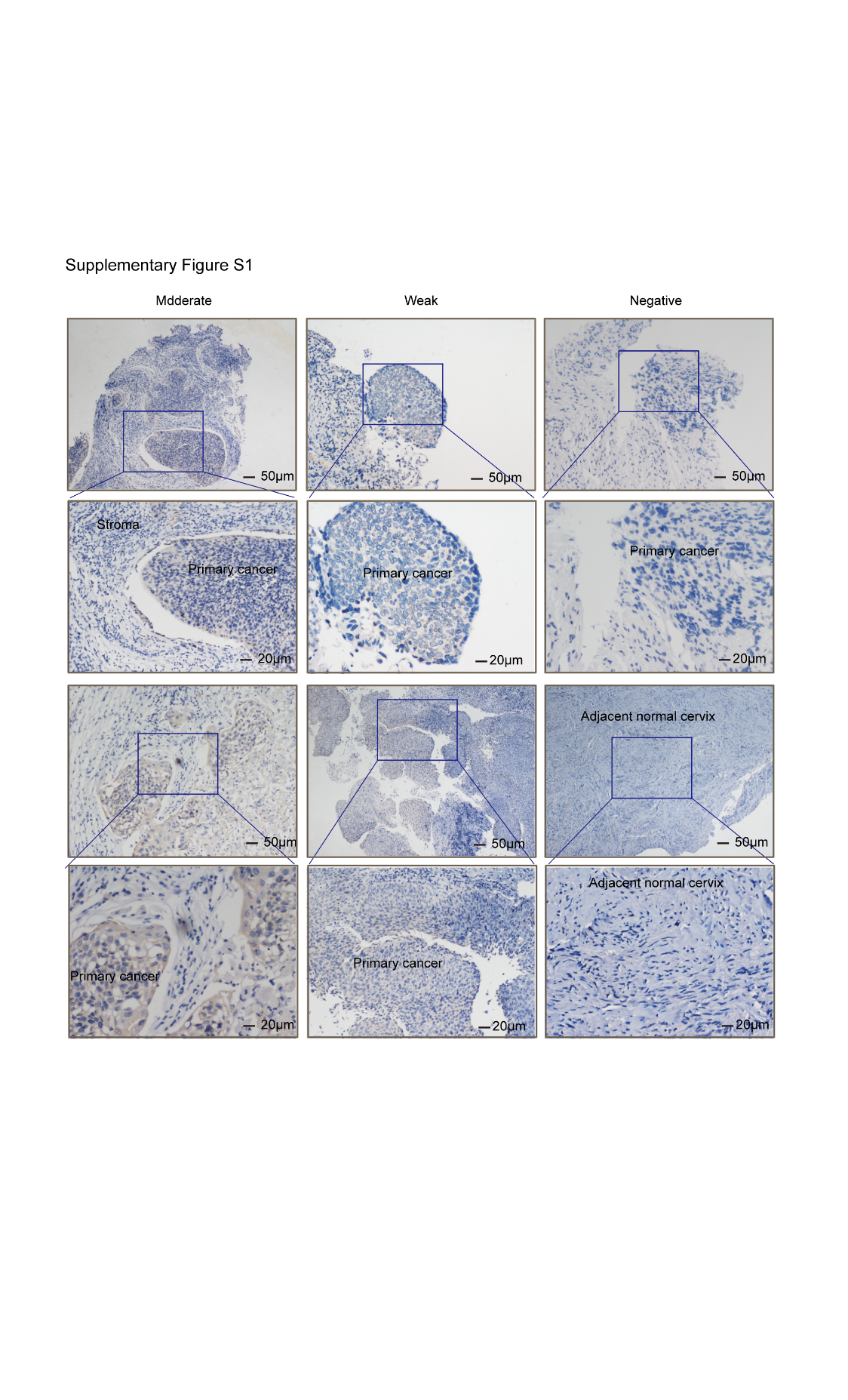


**Supplementary Figure S1: IHC staining status of LOXL2.** IHC staining status (negative, weak, moderate) of LOXL2 in samples.

**Supplementary Figure S2**


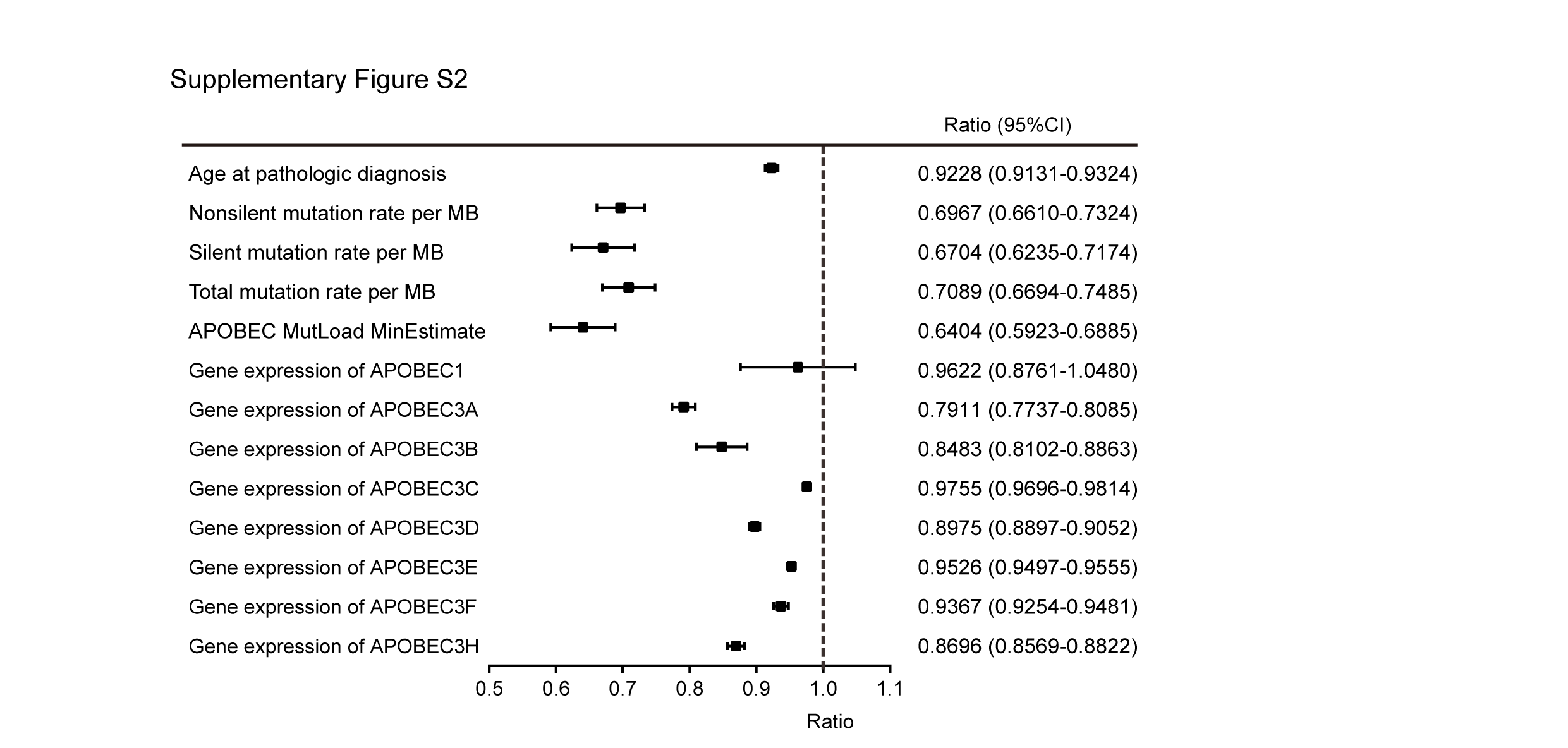


**Supplementary Figure S2: Forest plot of the correlation.** A forest plot with diagnostic age, and the expression of APOBEC3 family genes as APOBEC3A, APOBEC3B, APOBEC3C, APOBEC3D, APOBEC3E, APOBEC3F, APOBEC3G and APOBEC3H between the two groups.

**Supplementary Figure S3**


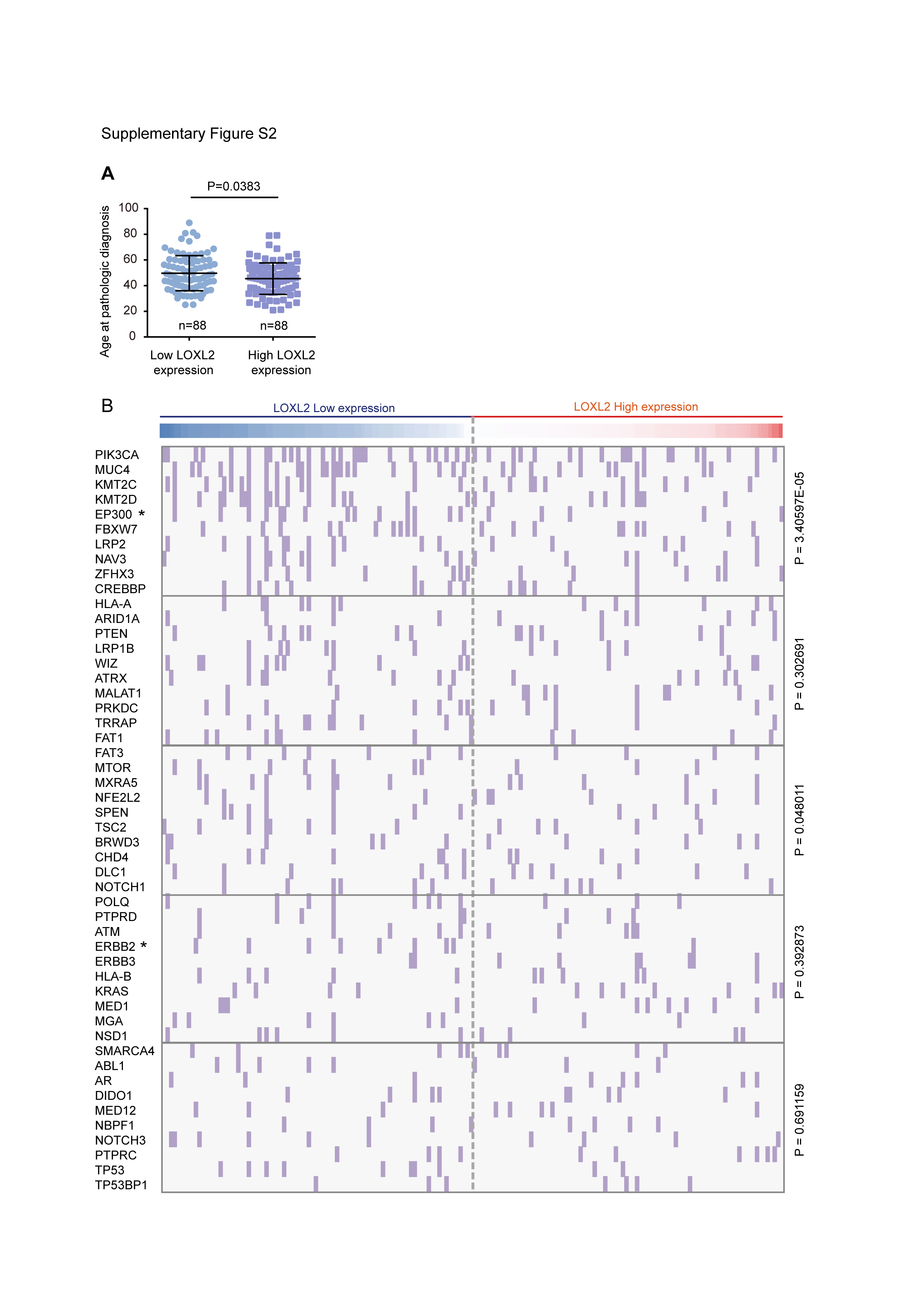


**Supplementary Figure S3: The mean diagnostic age and tumor mutation density were high in the LOXL2 low expression group.** (**A**) Comparison of diagnostic age in the low LOXL2 expression group with that of high LOXL2 expression group using 176 core-set cervical cancer samples. P values were calculated by Mann-Whitney test. (**B**) 176 core-set cervical cancer samples (columns) arranged by LOXL2 expression, top1-10, top11-20, top21-30, top31-40, and top41-50 somatic genomic alteration genes (rows) of patients in cervical cancer were shown. Each light purple column represents a somatic genomic alteration. * means statistical significance (<0.05). P values were calculated by Chi-square test.

**Supplementary Figure S4**


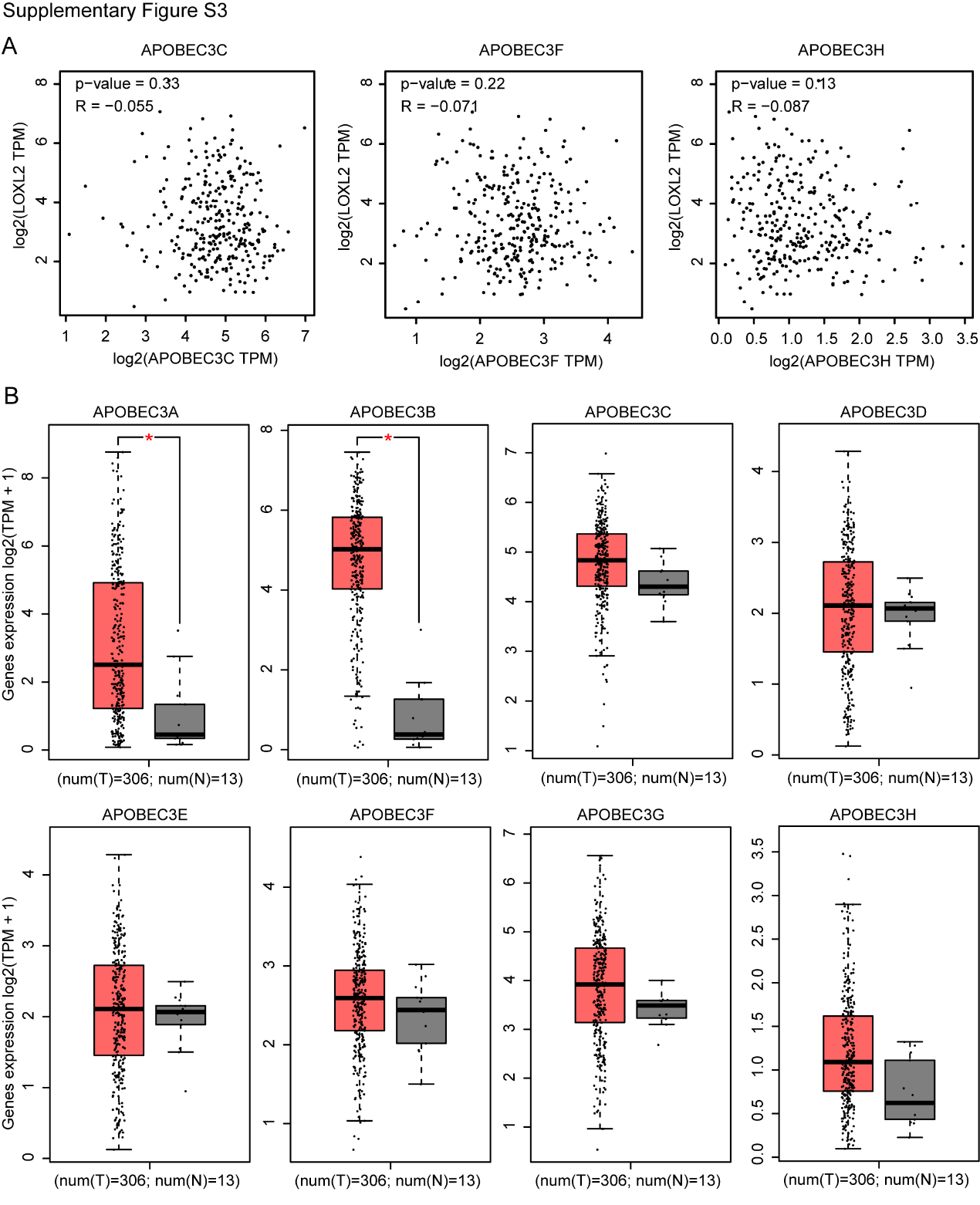


**Supplementary Figure S4: The correlation analysis of LOXL2 and APOBEC3 genes and The expression of APOBEC3 family genes in normal and tumor.** (**A**) Pearson correlation analysis of the expression of APOBEC3C, APOBEC3F and APOBEC3H with that of LOXL2 expression in TCGA dataset from GEPIA. P value and Pearson correlation coefficient (r) were shown. (**B**) The expression of APOBEC3 family genes as APOBEC3A, APOBEC3B, APOBEC3C, APOBEC3D, APOBEC3E, APOBEC3F, APOBEC3G and APOBEC3H in normal cervix (n=13) and cervical tumor (n=306). P value was calculated by one-way ANOVA. * means P < 0.05.

**Supplementary Figure S5**


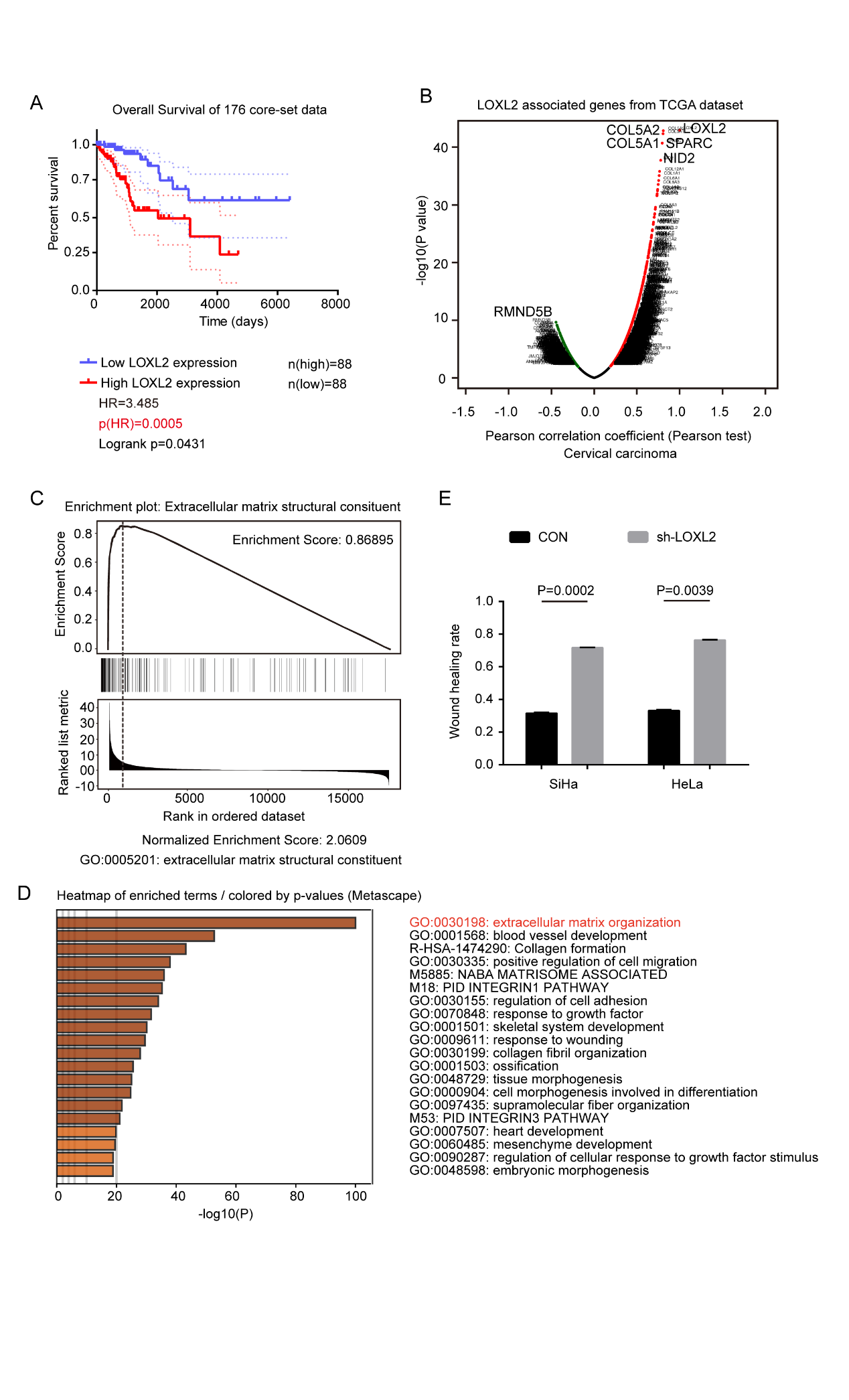


**Supplementary Figure S5: The expression of LOXL2 was associated with extracellular matrix organization in cervical cancer.** (**A**) Overall survival were compared between the LOXL2-high and -low groups corresponding to LOXL2 expression in 176 core-set samples. (**B**) LOXL2 correlated genes in cervical cancer from TCGA dataset were performed by LinkedOmics. (**C**) Enrichment plot concerning GSEA analysis of LOXL2 correlated genes. (**D**) Heatmap of enriched terms about LOXL2 associated genes were performed by Metascape (23). (**E**) Wound healing rate in the groups of SiHa and HeLa cell lines.

**Supplementary Figure S6**


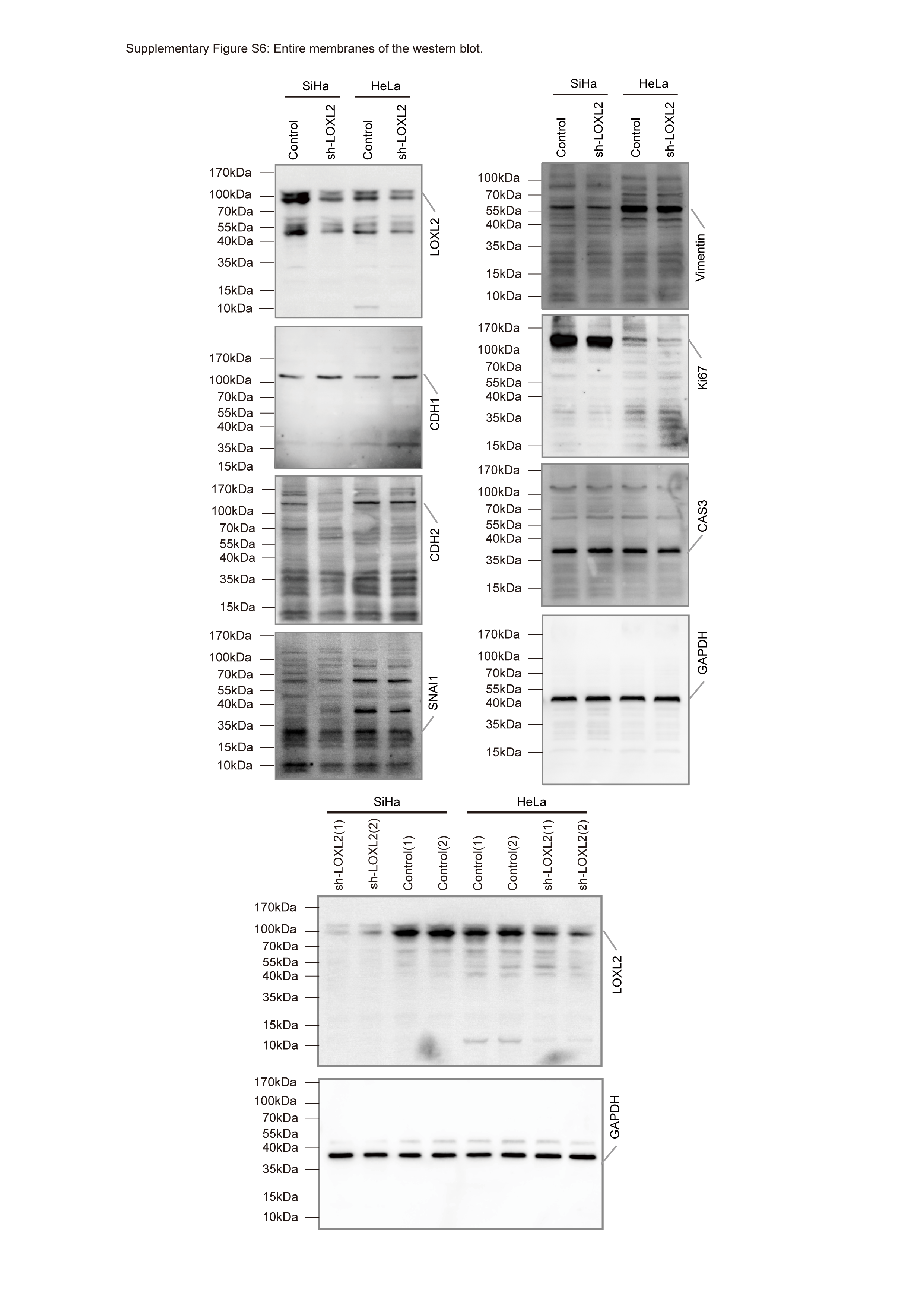


**Supplementary Figure S6: Entire membranes of the western blot.**
